# Factors associated with anxiety in colorectal cancer survivors: a scoping review

**DOI:** 10.1007/s11764-024-01678-0

**Published:** 2024-10-02

**Authors:** Juehyun Shin, Jessie S. Gibson, Randy A. Jones, Katrina J. Debnam

**Affiliations:** School of Nursing, University of Virginia, Charlottesville, VA USA

**Keywords:** Anxiety, Colorectal cancer, Risk factor, Consequence, Anxiety measure, Scoping review

## Abstract

**Purpose:**

Anxiety is one of the most common psychological issues among colorectal cancer (CRC) survivors. It can interact with physical symptoms, impacting cancer progression, survival, and quality of life. This scoping review aims to explore the factors associated with anxiety in patients with CRC and the instruments used to measure anxiety.

**Methods:**

Using Arksey and O’Malley’s (2005) framework for the scoping review, studies investigating anxiety in CRC patients published in CINAHL, PubMed, PsycINFO, and Scopus between 2013 and 2024 were included.

**Results:**

We analyzed fifty-one studies for this review. The review identified several risk factors and consequences of anxiety in CRC patients. The risk factors were classified into six domains using Niedzwiedz et al.'s (2019) framework: individual characteristics, social/ contextual factors, prior psychological factors, psychological responses to diagnosis and treatment, characteristics of cancer, and treatment. The consequences of anxiety were classified into three categories: global health status/quality of life, functions, and symptoms/problems. The most frequently used tool was the Hospital Anxiety and Depression Scale, with International Classification of Diseases codes being the second most used.

**Conclusions:**

This scoping review highlighted the intricate interaction between biological and psychosocial aspects in the lives of CRC survivors. It also identified unique factors associated with anxiety among these individuals. However, the review found some inconsistencies in the results related to anxiety-related factors, potentially due to differences in study populations, designs, measurement tools, and analysis methods.

Implications for Cancer Survivors.

This review underscores the potential for interventions targeting modifiable factors to prevent or reduce anxiety and enhance the quality of life for CRC survivors.

## Introduction

Colorectal cancer (CRC) is a common diagnosis, ranking as the third most common cancer in the US [1]. The American Cancer Society [2] estimates that in 2024, there will be 106,590 colon and 46,220 rectal cancer cases diagnosed in the US, with an expected 53,010 deaths from CRC. However, due to advances in early diagnosis and treatment, CRC-related mortality rates have been gradually declining [1]. Notably, the rate of young-onset CRC, diagnosed before the age of 55, has been increasing over the past two decades for unknown reasons [2, 3]. According to the American Cancer Society report, since the mid-1990s, the incidence of CRC has risen by 1% to 2% annually in individuals under 55 [2]. As a result, a growing number of survivors are expected to live longer while dealing with the effects of their disease and treatment, such as increased levels of anxiety.

According to a recent meta-analysis, the pooled prevalence of anxiety symptoms in CRC patients was 18.9% [4]. Excessive anxiety in any cancer patient is concerning as it is associated with increased disease complications, treatment issues, and mortality rates. A US-based prospective cohort study involving 1228 health professionals diagnosed with CRC found that increases in anxiety symptoms correlated with a 17% higher mortality risk, even after controlling for cancer characteristics [5]. Therefore, understanding the factors associated with anxiety within the CRC population is crucial for its effective prevention and treatment.

Despite numerous recent findings in this research area, no scoping review specifically focusing on anxiety in the CRC population has been published. Some systematic [6, 7] or literature reviews [8] exist, though these address narrower research questions such as the onset [6] or prevalence of anxiety [8], or the predictive value of anxiety on the mortality of patients with CRC [7]. However, these reviews often exclude findings that would provide a broader view of this phenomenon. In addition, while the existing literature contains several comprehensive review articles on anxiety in cancer patients as a generalized group [9–14], there are few that specifically address anxiety in CRC patients. Synthesizing findings on anxiety in specific types of cancer is crucial, as each cancer type exhibits unique characteristics in terms of disease progression and treatment modalities. CRC patients often experience unique anxiety-related factors that differentiate them from other cancer patients, such as social isolation resulting from bowel or ostomy problems [15, 16]. The primary purpose of this scoping review is to synthesize and compile findings on the factors associated with anxiety in CRC patients. By analyzing the current literature, our goal is to identify risk factors and consequences of anxiety in CRC survivors, as well as the instruments used for assessment. Through this, we aim to pinpoint the current research gaps concerning anxiety in this population.

## Methods

We followed Arksey and O'Malley's [17] methodological framework for this scoping review. The research process was initiated with an initial article search in November 2023, in consultation with a health sciences librarian. To ensure that our research was up to date and included the most recent information available, we conducted a final follow-up search in April 2024. We utilized four key databases for the search: CINAHL, PubMed, PsycINFO, and Scopus. Our search strategy was carefully crafted to capture a broad range of articles related to anxiety and colorectal cancer. The specific query used was: ((anxiety[Title/Abstract]) AND (colorectal[Title/Abstract] OR colon[Title/Abstract] OR bowel[Title/Abstract] OR rectal[Title/Abstract])) AND (cancer[Title/Abstract] OR neoplasm*[Title/Abstract] OR carcinoma*[Title/Abstract] OR tumor*[Title/Abstract]).We included articles with ‘colorectal cancer’ and ‘anxiety’ keywords in their title or abstract, published in English between 2013 and 2024. We limited our search to this period to collect the most current research on this issue. We excluded studies where CRC or anxiety was not the major subject (e.g., mixed results with other cancer populations or other mental health outcomes like depression), those solely evaluating specific intervention aspects (e.g., surgical technique, cognitive behavior therapy), or studies centered on colorectal cancer screening.

**Fig. 1:**
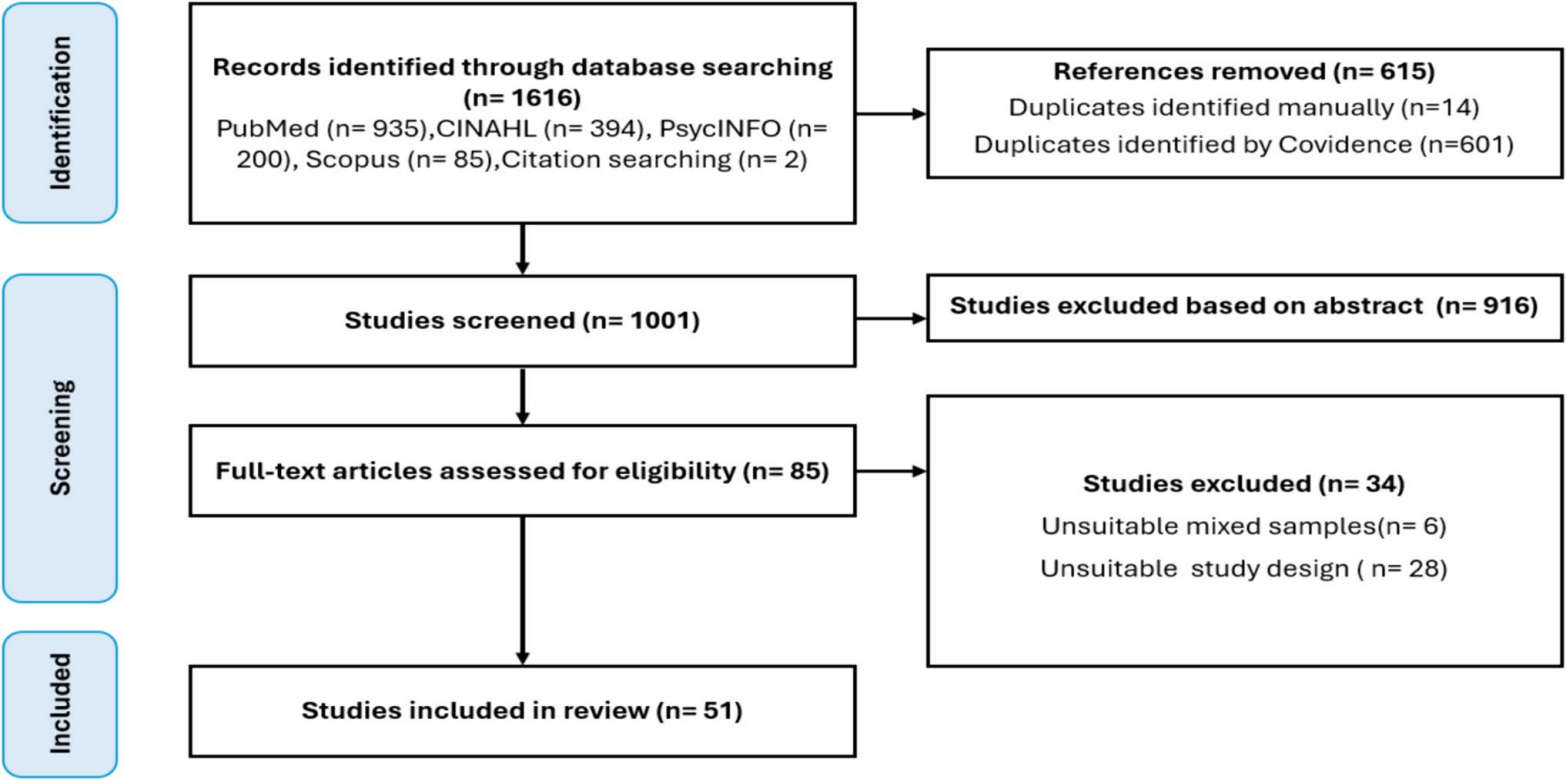
PRISMA for anxiety among colorectal cancer survivors

Our selection process is visualized using a PRISMA (Preferred Reporting Items for Systematic Reviews and Meta-Analyses) flow diagram (see Fig. 1). At the initial stage of our process, a total of 1616 studies were identified as potential sources for our review. Using Covidence, we identified 601 duplicate studies to remove from our pool. We also manually identified an additional 14 duplicates, further refining our pool. After eliminating these duplicates, we were left with 1001 studies. After reviewing titles and abstracts, we excluded 916 studies that did not meet our prespecified criteria for inclusion. We then reviewed the full text of the remaining 85 studies, leading to the exclusion of 34 additional studies. The reasons for their exclusion varied, including issues such as unsuitable mixed samples (e.g., results related to anxiety were mixed with those of other cancer populations or individuals without a cancer diagnosis) or unsuitable study design (e.g., results related to anxiety were combined with other mental illnesses such as depression, or studies where anxiety is not the focus) (Fig. 1). After this comprehensive review and exclusion process, 51 studies met our specified criteria and were included in our review.

The data charting process for this scoping review involved extracting key information from each included study. The extracted data items included the authors and year of publication, study design, the country where the study was conducted, cancer site, stage of cancer at the time of the study, sample size, exclusion of participants with a mental health history, study timeframe, measures used to assess anxiety, cut-off points used in these measures, identified risk factors for anxiety, and the consequences of anxiety as reported in the study.

## Results

The included articles comprise three review papers, 43 quantitative studies (including 22 cross-sectional and 21 longitudinal studies), four qualitative studies, and one mixed-method study. These studies were conducted in various countries, with the majority in the US (n = 12) and China (n = 10), including Brazil, Spain, the Netherlands, and Japan. The characteristics of the included studies are summarized in Table 1. In addition, this table categorizes variables into two groups: risk factors and consequences of anxiety. We consider independent variables as potential risk factors and dependent variables as potential consequences. We also consider timely sequences or mechanistic plausibility, such as gender being a risk factor of anxiety, not the other way around. The interpretation of results within the studies also significantly influences our classification process. However, it is important to note that due to the observational nature of most included studies, our ability to confidently establish a clear cause-and-effect relationship, or even directionality, between anxiety and the other variables is inherently limited.

**Table 1:** Included study characteristics, anxiety risk factors, consequences and assessment instruments

Authors (Year)	Study Design	Country	Cancer site	Cancer Stage	Sample Size	Exclusion of Participants with Mental Health History	Time	Anxiety Measures	Cut-off Points	Risk Factors	Consequences
Akyol et al. (2015) [18]	Cross-sectional	Turkey	Colon, rectum	N/A	105	N/A	N/A	HADS	Turkey version: ≥ 10 high anxiety	> Individual characteristics: female ( +)	Global QOL (-), physical functioning, role functioning, cognitive functioning, emotional functioning, and social functioning (-), symptoms (Fatigue, Nausea/Vomiting, pain, appetite loss) ( +), (Dyspnea, insomnia, constipation, diarrhea) (0), financial problems ( +), sexual (touch, avoidance, Anorgasmia) problems ( +)
Benedict et al. (2016) [19]	Cross-sectional	US	Rectal or anal cancer	I-III	70 (females)	N/A	Post-treatment & Mean years since treatment (SD) = 4.3 (3.3)	Brief Symptom Inventory (BSI) Anxiety subscale	continuous	> Prior psychological factors: body image disturbance ( +) > Characteristics of cancer: GI symptoms (e.g., feeling bloated, gas, and pain in abdomen) ( +), diarrhea (0) > Cancer treatment: having ostomy ( +)	N/A
Benedict et al. (2018) [20]	Cross-sectional	US	Rectal or anal cancer	I-III	144	N/A	Post-treatment & Mean years since treatment (SD) = 4.6 (3.3)	Brief Symptom Inventory (BSI) Anxiety subscale	continuous	> Individual characteristics: female ( +) > Characteristics of cancer: diarrhea ( +). diarrhea → social function (-)	N/A
Boehmer et al. (2021) [21]	Cross-sectional	US	Colon, rectum	I-III	Heterosexual (n = 353) and Sexual minority (n = 127)	N/A	Within 5 years following CRC diagnosis	Health-related anxiety in QLQ-CR29, HADS	Health-related anxiety > dichotomized: < 51 vs. ≥ 51 HADS > Dichotomized < 8: normal ≥ 8: presence of anxiety	> Individual characteristics: Sexual minority ( +)	N/A
Boehmer, Clark, et al. (2022) [22]	Cross-sectional	US	Colon, rectum	I-III	Sexual minority (n = 127)	N/A	Within 5 years following CRC diagnosis	Health-related anxiety in QLQ-CR29, HADS	Health-related anxiety > dichotomized: < 51 vs. ≥ 51 HADS > continuous	> Individual characteristics: Age at diagnosis (-,0), sexual minority identity in years (-) > Social & Contextual factors: discrimination ( +), employed ( +), neighborhood poverty ( +) > Psychological response to diagnosis and treatment: denial coping ( +), embarrassment due to stoma or bowel movements ( +) > Characteristics of cancer: dry mouth ( +)	N/A
Boehmer, Ozonoff, et al. (2022) [23]	Cross-sectional	US	Colon, rectum	I-III	Heterosexual (n = 353) and Sexual minority (n = 127)	N/A	Within 5 years following CRC diagnosis	Health-related anxiety in QLQ-CR29, HADS	Health-related anxiety > dichotomized: < 51 vs. ≥ 51 HADS > Continuous	> Individual characteristics: Sexual minority after adjusting for covariates (0), age at diagnosis (-,0), poor health ( +), comorbidity ( +), overweight or obese (-) > Social & Contextual factors: employed ( +), discrimination experience ( +), loneliness ( +), education level (-), support (-) > Psychological response to diagnosis and treatment: attending support group ( +), active coping ( +), low body image ( +), weight concerns ( +), resilience (-) > Characteristics of cancer: Abd pain ( +), dry mouth ( +), embarrassment ( +), flatulence ( +) > Cancer treatment: chemo ( +)	N/A
Bonhof et al. (2019) [24]	Cross‐sectional	Netherlands	Colon, rectum	I—IV	1643	N/A	Mean years since diagnosis (SD) = 6.1 (2.8)	HADS	Continuous	> Cancer treatment: Chemotherapy-Induced Peripheral Neuropathy ( +)	Fatigue ( +)
Braamse et al. (2016) [25]	Cross-sectional	Netherlands	Colon, rectum	I—IV	91	N/A	Diagnosed 3.5 to 6 years ago	Beck Anxiety Inventory	Continuous	> Individual characteristics: comorbidity ( +) > Characteristics of cancer: The time since cancer diagnosis (-)	N/A
Carlile and McAdam (2023) [16]	Qualitative	UK	Colon, rectum	I–III (Duke classification: A-C)	15	N/A	Completed curative treatment at least 6 months previously	Interview	N/A	> Psychological response to diagnosis and treatment: fear of recurrence ( +) > Cancer treatment: bowel or ostomy dysfunction ( +)	Social functioning (-), sexual dysfunction, dietary changes
Cheng et al. (2022) [6]	Systematic Review and Meta-Analysis	N/A	Colon, rectum	N/A	8 cohort studies (only 6 studies assessed anxiety)	N/A	The included articles were published between 2010 and 2021	ICD-9 code (n = 7), Hospital Anxiety and Depression Scale (HADS) (n = 1)	N/A	> Individual characteristics: Age at diagnosis ( ±), female ( +), comorbidities ( +) > Characteristics of cancer: cancer stage ( +), cancer site (0: CRC diagnosis, no matter the site, is linked with increased anxiety) > Cancer treatment: radiotherapy ( +), chemotherapy ( +), colostomy ( +)	After adjusting for age, sex, and number of comorbidities: global QOL (-), physical functioning, role functioning, cognitive functioning, emotional functioning, and social functioning (-) mortality ( +)
Di Cristofaro et al. (2014) [26]	Prospective Cohort	Italy	Colon, rectum	I—IV	116	N/A	At the time of admission and at 1 and 6 months after surgery	EORTC colorectal cancer module (CR29)	Continuous	> Cancer treatment: 1 month after surgery, complications ( +)	N/A
Gonzalez-Saenz de Tejada et al. (2016) [27]	Longitudinal	Spain	Colon, rectum	N/A	972	N/A	Before surgery and 12 months afterwards	HADS	< 8: Non-case ≥ 8: Borderline case ≥ 11: Probable case	N/A	After adjusting for age, location, gender, and baseline HRQoL: emotional functioning (-), physical functioning, role functioning, cognitive functioning, emotional functioning, and social functioning (0), pain ( +), fatigue ( +)
Gonzalez-Saenz de Tejada et al. (2017) [28]	Longitudinal	Spain	Colon, rectum	0—IV	947	N/A	Before surgery and 12 months afterward	HADS	Continuous	> Individual characteristics: age (-), female ( +), married (0) > Social & Contextual factors: Unemployed (0), social support (-), > Psychological response to diagnosis and treatment: baseline HADS (-), insomnia ( +) > Characteristics of cancer: cancer stage (0), location (0), physical function (-), cognitive function (-), social function (-), functionally independent (-)	N/A
Gray et al. (2014) [29]	Cross-sectional	UK	Colon, rectum	N/A	496	N/A	Within 26 weeks or between 48 weeks to 2 years since CRC diagnosis	HADS	Dichotomized < 8: non-case ≥ 8: borderline case	> Individual characteristics: smoker ( +) > Social & Contextual factors: unemployed ( +), living in a deprived area ( +), difficulty in carrying out domestic chores, financial difficulty ( +), communication difficulty ( +), living arrangement difficulty ( +), isolation ( +) > Prior psychological factors: Hx Anxiety or Depression ( +) > Psychological response to diagnosis and treatment: negative emotional consequences ( +), depression ( +) > Characteristics of cancer: Nausea or vomiting ( +), dyspnea ( +), sleep disturbance ( +), diarrhea ( +), pain ( +), # of symptoms ( +), cognitive functioning (-)	N/A
Hess et al. (2023) [30]	Cross-sectional	US	Colon, rectum	I—IV	277 (≥ 60 years)	N/A	N/A	Patient-Reported Outcomes Measurement Information System (PROMIS) Anxiety Four-item Short Form	Dichotomized as Not or mild anxiety (t-score < 60) vs Moderate or severe anxiety (t-score ≥ 60)	> Individual characteristics: age (0), female (0), race-ethnicity (0), married (0) > Social & Contextual factors: educational level (0), unemployed (0) > Characteristics of cancer: cancer stage (0)	After adjusting age, sex, race, and cancer stage: depression ( +), frailty ( +), physical HRQOL (-), mental HRQOL (-)
Holthuijsen et al. (2024) [31]	Prospective Cohort	Netherlands	Colon, rectum	I-III	249	N/A	At 6 weeks, 6 months, 12 months, 24 months, and 60 months post-treatment	HADS	< 8: No anxiety disorder ≥ 8: Potential anxiety disorder	> Characteristics of cancer: After adjusting confounder, plasma 3-hydroxyanthranilic acid (-), Time after post-treatment (0)	N/A
Howren et al. (2022) [32]	Retrospective Cohort	Canada	Colon, rectum	N/A	54,634 (&546,340 cancer-free)	Met the case definition for anxiety before the index date	Initial (12 months after diagnosis), continuing (Between initial and end of life phase), end-of-life (12 months before cancer death)	ICD codes (9 or 10): ICD-9: 300.0, 300.2 ICD-10: F40-F41	To meet the case definition for anxiety, ≥ 1 inpatient ICD code or 2 outpatient ICD codes within a 2-year period	> Individual characteristics: In participants diagnosed with CRC under the age of 50, the risk of anxiety was similar to those of cancer-free controls (0), Participants diagnosed with CRC after the age of 50 had a higher risk of anxiety than cancer-free individuals in the same age group ( +), after adjusting for the confounding variables, age at diagnosis (0) > Characteristics of cancer Time since CRC diagnosis (b/w initial and end of life > 12 months after CRC diagnosis > 12 months before cancer death)	N/A
Hu et al. (2022) [33]	Case–Control	China	Colon, rectum	I-III	602	Mental disease or pre-operate HADS > = 8	Pre-surgery and 2 weeks post-discharge	HADS	Dichotomized: < 8: Normal ≥ 8: Anxiety	> Individual characteristics: female ( +), age (0), married (-), comorbidity ( +) > Social & Contextual factors: education level (0), dissatisfaction with income ( +) > Characteristics of cancer: Cancer stage (0), Poor performance Status ( +) > Cancer treatment: Postoperative complication ( +), chemotherapy ( +), permanent stoma ( +)	N/A
Huang et al. (2023) [34]	Prospective Case–control	China	Colon, rectum	I—IV	362	Mental illness	1–2 days before and after chemo	HADS	Continuous	N/A	Cancer-related fatigue ( +)
Hyphantis et al. (2016) [35]	Prospective Cohort	Greece	Colon, rectum	I-III	84 (& 82 breast cancer, 50 unknown cancer, and 84 healthy controls)	History of psychotic illness	Baseline and after one year & Mean months since diagnosis (SD) = 15.5 (23)	Symptom Distress Check List (SCL-90-R)	Continuous	> Characteristics of cancer Time ( +)	Trouble falling asleep ( +), wakening up early in the morning (0)
Jakobsson et al. (2016) [36]	Prospective Cohort	Sweden	Colon, rectum	N/A	105	N/A	Pre-surgery and up to 6 months post-surgery	State-Trait Anxiety Inventory (STAI)	Continuous	> Characteristics of cancer: Time, before surgery ( +) > Cancer treatment: Types of CRC surgery: rectal resection, abdominoperineal resection, and colonic resection (0) Within the groups (colonic resection reported less anxiety at discharge than before surgery)	N/A
Jin et al. (2019) [37]	Longitudinal	China	Rectal cancer	I—IV	67	History of mental illness	1–2 days pre-surgery (with colostomy) and pre-discharge	HADS	Continuous	> Characteristics of cancer: pre-surgery ( +)	Psychosocial behavior reactions (-): impact, acknowledgment, retreat, and reconstruction
Kerckhove et al. (2021) [38]	Cross-sectional	France	Colon, rectum	N/A	96	N/A	≤ 5 years from the time chemotherapy was discontinued & who were treated with adjuvant oxaliplatin-based chemotherapy	HADS	< 8: Normal ≥ 8: Borderline or suggestive of anxiety ≥ 11: Indicative of anxiety	> Cancer treatment: Chemotherapy-Induced Peripheral Neuropathy ( +)	N/A
Lim et al. (2022) [39]	Mixed method (cross-sectional & semi-structured interviews)	Australia	Colon, rectum	IV	38	N/A	Between 0.5–2 years post-surgery. Mean months since treatment (SD) = 14 (5)	interview	N/A	> Psychological response to diagnosis and treatment: active confrontation (-), meaning making (-), and acceptance (-), active avoidance (-, +), passive avoidance (-, +)	N/A
Lloyd et al. (2019) [40]	Retrospective Cohort	US	Colon, rectum	I—IV	8,961	Prior history of mental illness	At 0–2 years, > 2–5 years or > 5 years after CRC diagnosis	ICD-9 diagnostic codes	Clinical classifications software	> Characteristics of cancer: The time since cancer diagnosis (-) (Adj. HR): 0–2 Years (2.84), 2–5 Years (1.24), and + 5 Years (1.3)	Mortality ( +), but this is a mixed result with other mental illnesses
Miranda et al. (2014) [41]	Cross-sectional	Brazil	Colon, rectum	N/A	20 (& 20 healthy)	Schizoaffective disorder, bipolar disorder, or panic disorder	Within 15 to 30 days after diagnosis and admitted for tumor resection	HADS	Continuous	> Characteristics of cancer: proinflammatory cytokine levels: IL-1β ( +), IL-6 ( +), IL8 ( +), TNF-α ( +), IL-10 (-)	N/A
Miranda et al. (2017) [42]	Cross-sectional	Brazil	Colon, rectum	N/A	80	Schizoaffective disorder, bipolar disorder, or panic disorder	Four groups (n = 20 each): pre-surgery; post-resection without therapy; on chemotherapy for ~ 3 months; and post ~ 6-month chemotherapy	HADS	Continuous	> Characteristics of cancer: Fractalkine serum levels ( +) at different stages of antitumor therapy	N/A
Miranda et al. (2018) [43]	Cross-sectional	Brazil	Colon, rectum	III	60 (& 20 healthy)	Schizoaffective disorder, bipolar disorder, or panic disorder	Pre-chemotherapy, Under chemotherapy and post-chemotherapy, n = 20 in each group	HADS	Continuous	> Characteristics of cancer: proinflammatory cytokine levels: IL-1β ( +), IL-6 ( +), IL8 ( +), TNF-α ( +), IL-10 (-) at different stages of antitumor therapy	N/A
Mohamed et al. (2021) [44]	Qualitative	US	Bladder, colon, or rectal	N/A	30 (& 13 caregivers)	N/A	Completed ostomy surgery Time since surgery: < 1 year 20% 1–2 years 50% > 2 years 30%	Interview	N/A	> Cancer treatment: Learning about stoma care by’ trial and errors’, need to master ostomy care, everyday challenges in the utility of stomal appliances, post-surgical morbidity, changes in body image, and difficulties resuming ‘normal activities’	N/A
Mols et al. (2018) [45]	Prospective Cohort	Netherlands	Colon, rectum	I—IV	2625 (& 315 cancer-free)	N/A	In 2010, 2011, 2012, and 2013 (data from the cancer registry) & mean years since diagnosis (SD) = 5.2 (2.8)	HADS	< 8: Low ≥ 8: High	> Individual characteristics: cancer group ( +), age (-), female ( +), married (-), comorbidities ( +) > Social & Contextual factors: education level (-) > Characteristics of cancer: Cancer stage (0), Time since CRC diagnosis (0) > Cancer treatment: Radiotherapy (0), Chemotherapy (0)	After adjusting for age, sex, and number of comorbidities: global QOL (-), physical functioning, role functioning, cognitive functioning, emotional functioning, and social functioning (-)
Orive et al. (2022) [46]	Prospective Cohort	Spain	Colon, rectum	0—IV	2531	Any severe mental conditions	Before surgery, and at 1, 2, 3, and 5 years after surgery	HADS	< 8: Absence of anxiety ≥ 8: Possible case ≥ 11: Higher a probable case	> Individual characteristics: female ( +) > Psychological response to diagnosis and treatment: baseline anxiety level ( +) > Characteristics of cancer: stage ( +) > Cancer treatment: chemotherapy ( +), Complications at 1 year after surgery ( +)	Mortality ( +)
Peng et al. (2019) [8]	Literature Review	N/A	Colon, rectum	N/A	15 cohort studies	N/A	The included articles were published between 1967 and 2018	HADS (7) ICD-9 (2) Minnesota Multiphasic Personality Inventory (1), Brief Symptom Inventory-18 (1)	N/A	> Individual characteristics: age (+ /0)	N/A
Renna et al. (2022) [47]	Cross-sectional	US	Colon, rectum	I—IV	88	N/A	Within 1–3 months after diagnosis and before undergoing adjuvant cancer treatment	Beck Anxiety Inventory (BAI)	Continuous	N/A	After adjusting stage, comorbidities, BMI, age, and sex: pain ( +), fatigue ( +), C‐reactive protein ( +)
Révész et al. (2022) [48]	Longitudinal	Netherlands	Colon, rectum	I—IV	910 (non-drinkers = 191 vs. drinkers of alcohol = 719)	N/A	At diagnosis and 3-, 6-, 12-, and 24-months post-diagnosis	HADS	Continuous & Dichotomized: < 8: Low anxiety ≥ 8: Clinical anxiety	> Individual characteristics: Alcohol consumption (-)	N/A
Salamonsen et al. (2016) [49]	Qualitative	Norway	Rectal	I–III	9	N/A	Diagnosed within the last 6 months and have completed primary surgical treatment	Interview	N/A	> Social & Contextual factors life course disruption	Patient-defined health care needs (emotional needs)
Saunders et al. (2021) [15]	Qualitative	US	Rectal	I–III	15 (&, 5 caregivers, 10 physicians.)	N/A	Completed treatment	Interview	N/A	> Cancer treatment: bowel or ostomy dysfunction (leak, noise, odor, hernia)	Not eating or drinking before meeting to control urgency, carrying extra clothes, scanning the nearest bathroom, and fearing leaving their homes
Selvy et al. (2020) [50]	Cross-sectional	France	Colon, rectum	N/A	406	N/A	Received adjuvant oxaliplatin-based chemotherapy within 5 years	HADS	< 8: Normal ≥ 8: Borderline or suggestive of anxiety ≥ 11: Indicative of anxiety	> Cancer treatment: Chemotherapy-Induced Peripheral Neuropathy ( +)	N/A
Song et al. (2020) [51]	Cross-sectional	China	Colon, rectum	0—IV	282	N/A	1 to 2 weeks post-surgery	HADS	≥ 9 possible cases of anxiety & continuous	> Psychological response to diagnosis and treatment: body image disturbance ( +) > Cancer treatment: having ostomy ( +) ostomy status (temporary or permanent) (0)	N/A
Soria-Utrilla et al. (2022) [52]	Prospective Cohort	Spain	Colon, rectum	I—IV	215	N/A	Before surgery, during admission, and at 1-, 6-, and 12-months follow-up	HADS (before surgery)	≥ 8: possible presence of anxiety ≥ 11: probable presence of anxiety	After adjusting for age, sex, and cancer stage, Preoperative malnourished status ( +)	After adjusting for age, sex, and cancer stage: Surgical complications (0), mortality (0)
Sun et al. (2020) [53]	Cross-sectional	China	Colon, rectum	I—IV	434	Coexisting mental disorders, use of psychotropic drugs 7 days before the survey	The morning before the day of surgery	Hamilton Anxiety Rating Scale	Continuous	N/A	Preoperative insomnia ( +)
Tamura (2021) [54]	Cross-sectional	Japan	Colon, rectum	I—IV	121	History of severe anxiety, depression, or mental illness	Diagnosed at least 6 weeks ago and have completed one course of chemotherapy	HADS	Continuous	> Individual characteristics: age (0), female (0), married (0), comorbidity (0) > Social & Contextual factors: education level (0), financial difficulty ( +), self-disclosure (-) > Psychological response to diagnosis and treatment: resilience (-) > Characteristics of cancer: poor appetite ( +), poor concentration ( +), pain ( +), fatigue ( +), metastasis (0), nausea (0), hair loss (0), peripheral neuropathy (0), Abd fullness (0), diarrhea (0)	QOL (-) depression ( +)
Trudel-Fitzgerald et al. (2018) [55]	Prospective Cohort	US	Colon, rectum	N/A	145 (from the Nurses' Health Study)	N/A	Within 4 years following CRC diagnosis (Follow up every 4 years for 20 years after CRC diagnosis)	Crown-Crisp Index (CCI)	Dichotomized < 4: low vs ≥ 4: high	N/A	Healthy lifestyles (physical activity, diet, BMI, alcohol, and tobacco consumption) (-)
Trudel-Fitzgerald et al. (2020) [5]	Prospective Cohort	US	Colon, rectum	0—IV	1228 individuals from Nurses' Health Study and 504 from Health Professional	N/A	Within 4 years following CRC diagnosis. Follow-up over 28 years	8-item Crown-Crisp Index (CCI) 7-item Generalized Anxiety Disorder (GAD-7) scale or anxiolytics use	Dichotomized (y/n) clinical anxiety	> Individual characteristics: female ( +), Prevalent cardiometabolic disease (+ , 0)	Mortality ( +)
Vallance et al. (2015) [56]	Cross-sectional	Canada & Western Australia	Colon	I—IV	180	N/A	Mean months since diagnosis (SD) = 18.8(4.4) & not currently undergoing any adjuvant therapy	Spielberger’s State Anxiety Inventory (SAI)	Continuous	> Individual characteristics: moderate-to-vigorous intensity physical activity (-), Sedentary time (0)	N/A
van Putten et al. (2016) [57]	Prospective Cohort	Netherlands	Colon, rectum	I-III	1375	N/A	In 2010, 2011, 2012 & Mean years since diagnosis (SD) = 5.3 (2.8)	HADS	Continuous	N/A	Moderate-to-vigorous physical activity (-)
Wang et al. (2024) [58]	Cross-sectional	China	Colon, rectum	I—IV	370	History of psychiatric illness	Patients undergoing postoperative adjuvant therapy	GAD-7	N/A	N/A	Cancer-related fatigue (0)
Xia et al. (2024) [7]	Systematic Review and Meta-Analysis	N/A	Colon, rectum	N/A	12 cohort studies	N/A	The included articles were published between 2013 and 2023	HADS (5), Crown-Crisp Index (1), Generalized Anxiety Disorder-7 (1)	N/A	N/A	In univariate analysis (Unadjusted HR), mortality ( +) In multivariate analysis (adjusted HR), mortality (0)
Xiangting et al. (2023) [59]	Cross-sectional	China	Colon, rectum	I—IV	175	Psychiatric disorders	4 days- 6 months after ostomy	HADS	Continuous	N/A	Supportive care needs: Physiology and daily living needs ( +), Psychological needs ( +), Sexual needs (0), Care and support needs ( +), Health information needs ( +)
Zhang et al. (2016) [60]	Prospective Cohort	China	Rectal cancer	I—IV	852	N/A	After the first treatment and 6 months later	HADS	< 11: No case ≥ 11: Clinical anxiety	> Prior psychological factors: Type D personality ( +)	N/A
Zhou & Sun (2021) [61]	Longitudinal	China	Colon, rectum	I-III	302 (who underwent resection)	N/A	At discharge and then every 3 months till Month 36	HADS	Dichotomized < 8: Non-case ≥ 8: Anxiety	Associated with 3-year anxiety risk, > Individual characteristics: female ( +), married (-), smoker (-) > Social & Contextual factors unemployed ( +) > Characteristics of cancer: stage ( +), Time since discharge after resection surgery ( +)	N/A
Zhu et al. (2020) [62]	Longitudinal (Retrospective)	China	Colon, rectum	I—IV	135	Concurrent psychiatric disorder or other mental problems	Starting time and completion time of chemotherapy	HADS	Dichotomized < 11: No ≥ 11: Case of anxiety	N/A	Early initiation of adjuvant chemotherapy (-)

0, Not statistically significant; + , Positive association; -, Negative association

*CRC* colorectal cancer, *HADS* hospital anxiety and depression scale, *global QoL* global health status and quality of life, *HRQOL* health-related quality of life, *N/A* not applicable

We classified data on risk factors into six domains using Niedzwiedz et al.’s [10] framework, designed to explain factors contributing to depression and anxiety among cancer patients. The framework considers individual characteristics, social/ contextual factors, prior psychological factors, psychological responses to diagnosis, characteristics of cancer, and treatment [10]. We modified the 'psychological responses to diagnosis' domain to 'psychological responses to diagnosis and treatment', as most psychological factors in our included studies were evaluated during or after treatment.

Based on data extracted from the included studies, we summarized the risk factors and consequences of anxiety in CRC survivors in Table 2. The consequences of anxiety in survivors of CRC were multifaceted and could be divided into three categories: global health status/quality of life, functions, and symptoms/problems. These categories were derived from the subcategories of the European Organization for the Research and Treatment of Cancer Quality of Life Questionnaire-C30 (EORTC QLQ-C30), a quality-of-life instrument for cancer patients [63, 64]. In this instrument, a high score for the global health status or quality of life represents a high quality of life. A high score on a functional scale indicates a high or healthy level of functioning. Lastly, a high score for a symptom scale or item indicates a high level of symptomatology or problems. We modified ‘symptom scales/items’ to 'symptoms/problems’. If studies had outcome variables not assessed by the EORTC QLQ-C30, we categorized them according to their closest relevance with items of this instrument under each category.

**Table 2:** Risk factors and consequences of anxiety in colorectal cancer survivors

Categories	Subcategories/ Factors
Risk Factors	
Individual Characteristics	Age (- [28, 45], +, 0 [, 32, 33, 54]) [6, 8] Sex: female (+ [5, 6, 18, 20, 28, 33, 45, 46, 61], 0 [30, 54]), sexual minority (+ [21], 0 [23]) Ethnicity (0 [30]) Marital status: married (- [33, 45, 61], 0 [28, 30, 54]) Comorbidities (+ [5, 6, 23, 25, 33, 45], 0 [5, 54]) Behavioral factors: alcohol consumption (- [48]), physical activity (- [56]), smoking (- [61], + [29]), overweight or obsess (- [23]), malnourished (+ [52])
Social/Contextual Factors	Education level (- [23, 45],0 [30, 33, 54]) Employment status: employed (- [29, 61], + [22, 23], 0 [30, 65]) Financial status: financial difficulty (+ [22, 29, 33, 54]) Social support: support (- [23, 65]), self-disclosure (- [54]), discrimination (+ [22, 23]), loneliness (+ [23]) Disruptive life events prior to cancer diagnosis (+ [49])
Prior Psychological Factors	Pre-existing psychiatric disorders: pre-existing anxiety or depression (+ [29]) Personality: type D-personality (+ [60])
Psychological Responses to Diagnosis and Treatment	Fear of cancer recurrence (+ [16]) Baseline anxiety level (+ [46]) Depression (+ [29]) Insomnia (+ [65]) Coping behavior: meaning-making (- [39]), acceptance (- [39]), support group (- [39], + [23]), active avoidance (-, +) [39], passive avoidance (-, +) [39], denial (+ [22]) Resilience (- [23, 54]) Body image disturbance (+ [19, 23, 51]) Embarrassment (+ [22, 23])
Characteristics of Cancer	Stage: cancer (+ [6]), cancer stage (+ [6, 46, 61],0 [30, 33, 45, 65]) Cancer Site (0 [6, 65]) Time since diagnosis or treatment (- [25, 36, 37, 40], + [35, 61], 0 [31, 45]) Physical symptoms: GI symptoms (+ [19]), diarrhea (0 [19], + [20]), abdominal pain (+ [23]), flatulence (+ [23]), dry mouth (+ [22, 23]) Functional decline: cognitive functioning (- [29, 65]), physical functioning (-[65]), social functioning (- [20, 65]), performance (- [33]), functionally independent (- [65]) Blood biomarkers: IL-1β (+ [41, 43]), IL-6 (+ [41, 43]), IL8 (+ [41, 43]), TNF-α (+[41, 43]), IL-10 (-[41, 43]), Fractalkine (+ [42]), 3-hydroxyanthranilic acid (- [31]))
Cancer Treatment	Treatment modality: radiotherapy (+ [6, 23, 33, 46],0 [45]), chemotherapy (+ [6, 23, 33, 46], 0 [45]), ostomy (+ [6, 19, 51]), permanent ostomy (+ [33], 0 [51]), resection site (0 [36]) Complications(+ [26, 33, 46]): chemotherapy-induced peripheral neuropathy (+ [24, 38, 50]), ostomy complications (+ [15, 16, 44])
Consequences	
Global Health Status/ Quality of Life (QOL)	Global QOL (- [6, 18, 30, 45, 54], 0 [27]) Mortality (+ [5, 6, 40, 46], 0 [7, 52])
Functioning	Physical functioning (- [6, 18, 45, 59], 0 [27]) Role functioning (- [6, 18, 45, 59], 0 [27]) Cognitive functioning (- [6, 18, 45], 0 [27]) Social functioning (- [6, 15, 16, 18, 45], 0 [27]) Emotional functioning (- [6, 15, 16, 18, 27, 45]) Sexual functioning (- [16, 18],0 [59]) Behavioral functioning: start adjuvant chemotherapy early (- [62]), alcohol (+ [55]), smoking (+ [55]), physical activity (- [57]), psychosocial behavioral responses (- [37])
Symptoms/ Problems	Pain (+ [18, 24, 27, 30, 34, 47]), Fatigue (+ [18, 24, 27, 30, 34, 47], 0[58]), Frailty (+ [18, 24, 27, 30, 34, 47]), Insomnia (+ [35, 53], 0 [18]), Nausea (+ [18]), Vomiting (+ [18]), Appetite loss (+ [18]), Dyspnea (0 [18]), Financial difficulty (+ [18]), C-reactive protein (+ [47])

0, Not statistically significant; + , Positive association; -, Negative association

### Risk factors associated with anxiety in CRC survivors

#### Individual characteristics

While a few studies found no significant connections between the burden of comorbid health conditions and anxiety [5, 54], the majority revealed that CRC survivors with more comorbid health conditions typically have higher levels of anxiety [5, 6, 23, 25, 33, 45]. A prospective cohort study in the US involving 1228 health professionals suggested a link between poorer cardiometabolic health and clinical anxiety levels in females with CRC [5]. However, this link was not observed in male participants [5].

Researchers have found that certain lifestyle and behavioral characteristics of CRC survivors are associated with anxiety levels. For example, alcohol consumption [48] and moderate-to-vigorous physical activity [56] were linked to lower anxiety levels. However, studies have yielded mixed results on the connection between smoking and anxiety levels [29, 61]. A cross-sectional study in the UK found that current smokers had higher odds of anxiety cases than those who never smoked [29]. Conversely, a longitudinal study in China discovered that current smoking was an independent factor associated with a lower risk of anxiety over three years [61]. Additionally, a cross-sectional study found that participants who were overweight or obese had lower anxiety [23]. A study in Spain evaluated the anxiety levels and nutritional status of CRC patients before surgery, revealing that those with high anxiety levels were more likely to be malnourished compared to others [52].

#### Social & contextual factors

Gray et al. [29] measured the social difficulties of 496 CRC survivors using the Social Difficulties Inventory (SDI). Their study explored the relationship between these social factors and the levels of anxiety. The findings showed that participants with more social difficulties were more likely to have anxiety. In addition to this study, numerous others (see Table 2) have demonstrated the influence of various social/contextual factors on anxiety among CRC survivors. For instance, employment status has mixed results: some studies found lower anxiety in employed survivors [29, 61], others found higher anxiety [22, 23], or no significant association [30, 65]. Lower anxiety was noted in those with social support [23, 65] and cancer self-disclosure [54]. Higher anxiety was linked to discrimination or loneliness [22, 23].

A qualitative study in Norway involving nine adults recently diagnosed with stage I–III rectal cancer offered more contextualized insights into anxiety in CRC survivors [49]. The participants reported that grief, anxiety, and depression triggered by life events before their cancer diagnoses had significantly more disruptive effects than their cancer itself. These events included the loss of close relatives or loved ones from illness or serving as long-term caregivers for their ill loved ones for years [49].

#### Prior psychological factors

Studies have found that the psychological factors of CRC survivors before their diagnosis influence their anxiety levels after diagnosis. In a UK-based cross-sectional study involving 496 CRC survivors, individuals who reported a history of anxiety or depression on a patient-reported co-morbidities questionnaire were significantly more likely to be classified as anxiety cases compared to those who reported no such history [29].

In Greece, a prospective cohort study involving 852 individuals diagnosed with rectal cancer yielded interesting findings [60]. The researchers used the 14-item Type D Personality Scale (DS14) to classify the participants' personality types. They discovered that patients identified as having a Type D personality, characterized by increased negative affectivity and social inhibition, experienced notably higher levels of anxiety at the time of diagnosis and six months later compared to those with a non-Type D personality [60].

#### Psychological response to diagnosis and treatment

Regardless of the cancer type, fear of recurrence is a common psychological response among survivors. Through interviews with CRC survivors who had completed treatment, Carlile and McAdam [16] reported that this fear, or the worry that something was “ not right” [16, p. 100], caused anxiety among the study participants.

Several longitudinal studies have investigated the correlation between participants' baseline anxiety before CRC surgery and their anxiety levels at follow-up. In Spain, Gonzalez-Saenz de Tejada et al. [28] assessed the anxiety levels of 947 CRC patients before surgery and one year afterward. They found that participants with higher anxiety levels prior to surgery reported a more significant decrease in anxiety levels after one year. Orive et al. [46] assessed the anxiety levels of 2531 CRC patients before surgery, and at one, two, three, and five years post-surgery. Study findings showed that participants with high anxiety levels before surgery reported high anxiety levels even after five years.

CRC survivors utilized a variety of coping strategies to handle the burden of cancer and its treatment. While many strategies helped to mitigate anxiety, some were associated with increased anxiety over the long term. For instance, meaning-making, acceptance, and participating in support groups were associated with reduced anxiety [39]. However, both active and passive avoidance, as well as denial, were associated with increased anxiety in the long term [22, 39]. Interestingly, a cross-sectional study involving 353 heterosexual and 127 sexual minority CRC survivors found that attending a cancer support group or using active coping strategies was associated with greater anxiety [23].

#### Characteristics of cancer

Research shows mixed results regarding the relationship between the time since diagnosis or treatment and anxiety. While some studies find no significant variation in anxiety over time [31, 45], others report either a decrease [25, 36, 37, 40] or an increase [35, 61] as time progresses. For instance, Howren et al. [32] conducted a retrospective cohort study in Canada and found that CRC survivors displayed the highest anxiety rate during the continuing phase, which is between 12 months after CRC diagnosis and 12 months before cancer death. On the other hand, Lloyd et al. [40] found the highest rates of anxiety within two years of a cancer diagnosis in their retrospective cohort study using the Utah population database. Additionally, a longitudinal study from China reported a consistent increase in anxiety rates from discharge to three months post-discharge [61]. In addition, a different longitudinal study in China revealed that the anxiety level among rectal cancer patients decreased 1–2 days before hospital discharge compared to 1–2 days before surgery [37].

In Brazil, Miranda et al. [41, 43] published consecutive study findings examining the association between proinflammatory cytokine levels and anxiety or depression. Their study findings revealed that higher levels of IL-1β, IL-6, IL8, or TNF-α were associated with higher levels of anxiety, while lower level of IL-10 was associated with higher levels of anxiety. Furthermore, Miranda et al. [42] found that Fractalkine serum level was also positively associated with anxiety level. Moreover, a prospective cohort study conducted in the Netherlands found that in confounder-adjusted linear mixed models, higher concentrations of 3-hydroxyanthranilic acid were associated with lower anxiety scores [31]. However, in sensitivity analyses, this association did not remain statistically significant after FDR adjustment.

#### Cancer treatment

Research has revealed a significant association between CRC survivors who have undergone radiotherapy, chemotherapy [6, 23, 33, 46]. or ostomy surgery [6, 19, 51] and increased anxiety levels. However, Mols et al. [45] found no significant association between radiotherapy or chemotherapy and anxiety levels in their prospective cohort study in the Netherlands. Regarding different types of ostomies, the results also vary. While Song et al. [51] found no significant difference in anxiety levels between participants with temporary or permanent ostomies, Hu et al. [33] found a higher risk of anxiety in participants with permanent ostomies compared to those with temporary ones. Additionally, a prospective cohort study in Sweden found no difference in anxiety levels among participants who had a rectal resection, abdominoperineal resection, or colonic resection, although those who underwent colonic resection reported less anxiety at discharge than before surgery [36]. Anxiety due to ostomy-related issues was frequently reported, particularly in qualitative studies [15, 16, 44]. Participants voiced worries about potential or existing ostomy complications such as leakage, noise, odor, or hernias during interviews. These issues presented daily challenges, impeding their ability to resume normal activities [15, 16, 44].

Several studies have examined the role of cancer treatment side effects and complications on anxiety levels. These studies found that participants who reported complications after CRC surgery experienced greater anxiety than those without complications [26, 33, 46]. Chemotherapy-Induced Peripheral Neuropathy (CIPN) is one of the most studied CRC treatment side effects. Participants with more severe CIPN also reported higher anxiety levels [24, 38, 50].

### Consequences of anxiety in CRC survivors

#### Global health status / quality of life

In multiple studies, it has been observed that a heightened level of anxiety is closely associated with poor global health status and reduced quality of life [6, 18, 30, 45, 54]. However, Gonzalez-Saenz de Tejada et al. [27] found no significant correlation between these variables in their longitudinal study in Spain.

#### Functioning

A cross-sectional study of 175 CRC survivors with ostomies in China discovered an association between higher anxiety scores and increased supportive care needs, such as physiology and daily living needs, psychological needs, and health information needs [59]. Although this study did not find a connection between anxiety and sexual needs, another cross-sectional study in Turkey and a qualitative study in the UK identified a link between anxiety and sexual functioning among CRC survivors [16, 18]. Furthermore, qualitative studies disclosed that participants' social or emotional functioning was negatively impacted due to their ostomy [15, 16]. During these interviews, participants mentioned limiting their food or drink intake before social activities due to worries about handling ostomy-related issues in public spaces.

A retrospective longitudinal study conducted in China found that participants with elevated anxiety levels were less likely to start adjuvant chemotherapy early (within 4 weeks after operations) [62]. In another prospective cohort study in the US involving healthcare providers diagnosed with CRC, it was discovered that individuals with anxiety often led unhealthy lifestyles, such as consuming alcohol or smoking [55]. Moreover, participants with high anxiety engaged in less physical activity [57]. A longitudinal study in China on 67 rectal cancer survivors undergoing colostomy surgery found that higher levels of anxiety significantly predict lower psychosocial behavioral responses in colostomy patients [37].

#### Symptoms/ problems

While Akyol et al. [18] found no connection between anxiety and insomnia, other studies have identified a significant association [35, 53]. For instance, Hyphantis et al. [35] found that CRC patients with increased anxiety after one year had more difficulty falling asleep, but there was no significant change in waking up early.

### Instruments used to assess anxiety

In the included studies, researchers used a variety of instruments to measure the participants' anxiety levels. The most frequently used instrument was the Hospital Anxiety and Depression Scale (HADS) [66], a self-reported tool that allows participants to assess their own anxiety and depression levels. In contrast, some studies used International Classification of Diseases (ICD) codes [67], which reflect anxiety disorders officially diagnosed by mental health specialists. Further information about each instrument is summarized in Table 3. Cutoff points used to determine the presence or severity of anxiety in included studies are summarized in Table 1. In each of the three review manuscripts, we only counted the usage of individual tools once, regardless of how often they appeared. This method was used to avoid multiple counts, particularly if the original studies from our research were also included in those review manuscripts.

**Table 3:** Anxiety measurement tools

Measure	Studies (*n*)	Interpretation of Scores (in Original Versions)	Items (*n*)
Hospital Anxiety and Depression Scale (HADS)-Anxiety [66]	31	0–7: Normal 8–10: Borderline abnormal (borderline case) 11–21: Abnormal (case)	7
International Classification of Diseases (ICD) codes 9 or 10 [67], or Anxiolytics use	4	N/A	N/A
Generalized Anxiety Disorder-7 (GAD-7) [68]	3	0–4: Minimal, 5–9: Mild, 10–14: Moderate, 15–21: Severe	7
Health-related anxiety in QLQ-CR29 [69]	3	1 (not at all)- 4 (very much)	1
Brief Symptom Inventory-18 (BSI-18) Anxiety subscale [70]	3	0 (no anxiety)- 24 (high anxiety)	6
Crown-Crisp Index (CCI) [71]	3	0 (no anxiety)- 16 (high anxiety)	8
Beck Anxiety Inventory (BAI) [72]	2	0–9: None, 10–18: Mild, 19–29: Moderate 30–63: Severe	21
Spielberger State-Trait Anxiety Inventory (STAI) [73] or Spielberger State Anxiety Inventory (SAI)	2	20 (absence of anxiety)- 80 (high level of anxiety)	state (20) + trait (20)
Hamilton Anxiety Rating Scale [74]	1	0–7: No or minimal 8–14: Mild anxiety 15–23: Moderate anxiety 24 or greater: Severe	14
Patient-Reported Outcomes Measurement Information System (PROMIS) Anxiety Four-item Short Form [75]	1	Raw score 4–20 T-score < 55: Normal, 55–60: Mild, 60–70: Moderate, ≥ 70: Severe	4
Symptom Distress Checklist (SCL-90-R) [76]	1	0 (not at all)-36 (extreme)	9
Minnesota Multiphasic Personality Inventory (MMPI) [77]	1	N/A	N/A

*N/A* not applicable

## Discussion

This scoping review illustrates the unique anxiety-related factors that specifically affect the CRC population, a perspective not commonly found in reviews focusing on the general cancer population. A recurring theme among included studies is the presence of anxiety associated with bowel or ostomy dysfunctions, common side effects of CRC treatments. Qualitative studies focusing on CRC survivors, particularly those with ostomies, consistently find that individuals frequently dealing with embarrassing situations due to ostomy complications exhibit increased anxiety [15, 16, 44]. These situations commonly stem from ostomy leakage, odor, or noise in public spaces, contributing to heightened anxiety about social participation. To cope, these individuals often restrict their food and drink intake before or during social events, or continually look for restroom facilities when out [15, 16]. This hyper-vigilance and resulting self-imposed dietary limitations contribute to their high anxiety levels. Ultimately, such anxiety can lead to social withdrawal, creating a vicious cycle that further intensifies anxiety [15, 20]. These findings align with studies conducted on individuals who have undergone ostomy surgery for various reasons, indicating a broad impact of this condition on mental health [78–80].

Our findings show that high levels of proinflammatory cytokines (IL-1, IL-6, IL-8, TNF-α), low levels of IL-10 [41, 43], and high fractalkine levels (defined as levels > 3mg/L) [42] are linked to increased anxiety in CRC patients. These findings support previous studies on neuroimmune interaction in the context of cancer [81–83]. Stress, tumor biology, and cancer treatments affect neurobiology through inflammation, influencing anxiety symptoms in cancer patients by linking sympathetic nerve activity and immune responses to tumor development [81, 82]. Further, anxiety can present as physical symptoms such as pain and fatigue [47] through these neuroimmune mechanisms, threatening the long-term health and physical functioning of CRC patients. Understanding these interactions would provide a more comprehensive view of the relationship between psychological and biological health in this population.

The HADS is a commonly used self-report questionnaire for measuring anxiety and depression in oncology and other health settings. This questionnaire was also predominantly used in our reviewed studies with the CRC population (see Table 3). However, as shown in Table 1, we observed that different articles used various cut-off points. While most studies dichotomized the subjects using a score of 8, Zhang et al. and Zhu et al. [60, 62] used a score of 11, and Song et al. [51] used 9. This variation in cut-off points could potentially lead to contrasting results when comparing the anxiety rate between different groups, as opposed to conducting a correlation analysis.

Anxiety, as determined by ICD codes, and as referenced in several studies [6, 8, 32, 40], could potentially lead to inconsistencies when comparing with other studies that utilize self-report questionnaires. The ICD codes are used to indicate that an individual has been clinically diagnosed with an anxiety disorder by a healthcare professional. This is in contrast to self-reported surveys where individuals themselves report their symptoms and experiences, which may or may not align with a clinical diagnosis.

In quantitative studies, excluding those with pre-existing clinical anxiety before a CRC diagnosis or conducting a longitudinal study that compares anxiety levels at different times can help determine whether the observed anxiety is directly related to the CRC diagnosis or its treatment. Understanding this is crucial. If anxiety is a by-product of CRC or its treatment, it can inform more targeted prevention interventions for this population. However, some cross-sectional studies we reviewed did not exclude pre-existing clinical anxiety cases before the CRC diagnosis [18–25, 29, 30, 38, 47, 50, 51, 56], making it difficult to pinpoint the root source of the anxiety. This lack of clarity could potentially skew the results and interpretations.

In line with previous review studies of CRC survivors [6, 8], this scoping review presents mixed results about the association between age and anxiety levels. This could be due to differences in study populations, designs, measurement tools, and analysis methods. Boehmer, Clark, et al. [22] and Boehmer, Ozonoff, et al. [23] examined anxiety and health-related anxiety levels across different age groups at diagnosis. In their study of sexual minority CRC survivors [22], they found higher anxiety levels and a greater likelihood of health-related anxiety in those diagnosed between 21–49 years, compared to those diagnosed at 65 years and older. However, the 50–64 years age group showed no significant differences in anxiety level or health-related anxiety compared to the oldest age group. In a separate study involving both heterosexual and sexual minority CRC survivors, Boehmer, Ozonoff, et al. [23] found that younger individuals, specifically those in the 21–49 year age group, were more likely to experience health-related anxiety, as assessed by the single item QLQ-CR29, compared to those aged 65 years and older. However, the association between age and anxiety, as assessed by the HADS, was statistically non-significant in this group. This discrepancy could be due to the different aspects of anxiety each tool measures. On the other hand, Howren et al. [32] categorized participants into three groups: young-onset CRC (diagnosed before 50 years old), average-age-onset CRC (diagnosed at 50 years or older), and cancer-free controls. They found that participants diagnosed with CRC under the age of 50 had a similar risk of anxiety to cancer-free controls in the same age group. However, those diagnosed with CRC after the age of 50 had a higher risk of anxiety than cancer-free individuals in the same age group. After adjusting for confounding effects such as current age, the differences in anxiety risk between CRC survivors diagnosed before 50 years and those diagnosed at or after 50 years were found to be statistically insignificant [32]. Unlike these studies [22, 23, 32], other included studies [28, 30, 33, 45, 54] utilized participants' current age at the time of the study to examine its association with anxiety levels. Some of these studies [30, 33, 54] found no significant link between age and anxiety levels, while others [45, 65] found an association. Hess et al. [30] only included CRC survivors who were 60 years or older (median age = 68), which might have led to insignificant differences in anxiety levels across different ages. Hu et al. [33] and Tamura [54] compared anxiety levels between those < 65 and ≥ 65, finding no significant differences in anxiety levels between the two age groups. However, Gonzalez-Saenz de Tejada et al. [65] found that older CRC survivors reported a more significant decrease in anxiety levels after a year of surgery. Mols et al. [45] discovered that older participants reported lower anxiety levels, aligning with findings from studies of the general cancer population [14, 84].

### Limitations

While this review is intended to be comprehensive, it does have limitations that are largely consistent with those of a scoping review design. We used study design, timelines, mechanistic plausibility, and interpretation to categorize variables as risk factors or consequences of anxiety. However, the observational nature of most studies, along with some cross-sectional designs, limits our ability to establish cause-and-effect relationships between anxiety and other variables definitively. Consequently, some variables were classified as risk factors in some studies and as consequences of anxiety in others. In addition, our scoping review identifies and maps factors associated with anxiety in CRC patients, but it does not provide an in-depth analysis of study quality and risk of bias, as systematic reviews typically do. Instead, it offers a broad overview of existing literature, identifying research trends, gaps, and future investigation areas. As an exploratory study, this scoping review may lack the rigor of systematic reviews due to its less strict inclusion criteria. However, this approach allows for a more inclusive and extensive literature examination, capturing a broader range of studies and perspectives. Furthermore, anxiety is a multidimensional concept that shares common characteristics with other emotional states like depression. In the context of cancer, anxiety is particularly linked to the fear of cancer recurrence and the fear of death, making it hard to distinguish from other emotional conditions. This complexity is a significant limitation of our study and should be considered when interpreting the results.

### Recommendations for future research

It is crucial to identify modifiable factors related to anxiety, as these factors could be the focus of future research on targeted prevention methods. Furthermore, understanding the complex relationships between these factors can provide a holistic view, enabling the implementation of early prevention strategies. While many qualitative studies have pinpointed psychosocial issues related to ostomy complications in the CRC population, few quantitative studies have investigated this. Benedict et al. [20] found a link between diarrhea and anxiety in survivors of rectal or anal cancer after treatment. However, it was not distinguished whether these individuals had an ostomy or not. In people with ostomies, diarrhea does not necessarily lead to ostomy leakage. Therefore, for future studies, we recommend using ostomy-specific questionnaires in quantitative research of CRC-related psychosocial outcomes. This will help investigate the correlation between ostomy complications and psychosocial problems among CRC survivors with ostomies.

## Conclusions

As the survival rates for CRC increase, understanding the potential biological and psychosocial effects of CRC and its treatments becomes increasingly critical. Our review significantly contributes to this understanding by identifying various predictors and outcomes of anxiety in this group. Modifiable factors present targets for interventions aimed at reducing anxiety and enhancing quality of life. Non-modifiable risk factors help pinpoint patients at high risk for early psychological support. Moreover, these risk factors can be further examined to comprehend their interactions.

## Data Availability

No datasets were generated or analysed during the current study.
